# Witnessing Cyberbullying and Internalizing Symptoms among Middle School Students

**DOI:** 10.3390/ejihpe10040068

**Published:** 2020-10-04

**Authors:** Diana M. Doumas, Aida Midgett

**Affiliations:** 1 Institute for the Study of Behavioral Health and Addiction, Boise State University, 1910 University Drive, Boise, ID 83725, USA; aidamidgett@boisestate.edu; 2 Department of Counselor Education, Boise State University, 1910 University Drive, Boise, ID 83725, USA

**Keywords:** bystander, cyberbullying, cross-sectional, between-group design, internalizing symptoms, middle school

## Abstract

Cyberbullying is a significant problem among school-aged youth. Cyberbullying peaks in middle school with 33% of middle school students reporting cyberbullying victimization and more than 50% reporting witnessing cyberbullying as bystanders. Although the association between cyberbullying victimization and internalizing symptoms is well documented, there is limited research examining the impact of witnessing cyberbullying on bystanders. To assess differences in internalizing symptoms between cyberbullying bystanders and non-bystanders, a school-based cross-sectional study was conducted among middle school students (6th–8th grade) in the United States (*N* = 130; 57.4% female; 42.6% male). Questionnaire data were analyzed using multivariate analysis of co-variance (MANCOVA) with three outcome variables (depression, anxiety, somatic symptoms) and the between-subject factor bystander status (bystander, non-bystander). We controlled for witnessing school bullying to examine the unique effect of witnessing cyberbullying on internalizing symptoms. Results of the MANCOVA indicated a significant effect for cyberbullying bystander status (*p* < 0.04). Post hoc analyses demonstrated that bystanders reported significantly higher levels of depression (*p* < 0.05), anxiety (*p* < 0.02), and somatic symptoms (*p* < 0.01) than non-bystanders. Findings suggest that programs to support students who witness cyberbullying are needed to reduce the mental health risks associated with being a cyberbullying bystander.

## 1. Introduction

Researchers have defined cyberbullying as a repeated, intentional act of aggression carried out by a group or individual, using electronic forms of contact, against a target who cannot easily defend him or herself [1]. Cyberbullying often takes place through e-mail, blogs, instant messages, text messages, chat rooms, websites, online games, or social networking sites [2]. National survey data from the United States indicate that cyberbullying peaks during middle school, with 33.0% of middle school students reporting being cyberbullied [3], although rates as high as 84% have been reported [4]. Although some researchers have suggested that cyberbullying is an extension of traditional face-to-face bullying [5], others argue that cyberbullying may be associated with greater harm than traditional bullying due to the potential for a large audience, unlimited access to targets, the possible anonymity of the cyberbully, and less adult supervision [6]. Findings from two recent meta-analyses [7,8] and a review of the cyberbullying literature [9] indicate that being a target of cyberbullying is associated with significant mental health risks including depression, anxiety, somatic symptoms, and suicidal ideation. Further, these negative mental health outcomes have been documented even when controlling for traditional bullying [10]. 

The consequences of cyberbullying are not limited to targets, but extend to students who witness cyberbullying as bystanders [11]. Bystanders may intervene in cyberbullying either directly (e.g., by telling the cyberbully to stop) or indirectly (e.g., by reporting the incident) [12], encourage the cyberbully (e.g., through commentary or like buttons) [7,13], join the cyberbully (e.g., forwarding texts or posts), or remain passive by doing nothing [13]. Research indicates 52.9% of middle school students report witnessing cyberbullying in the past six months [14] and peers play an important role in maintaining cyberbullying [15]. Thus, it is important to gain a better understanding of cyberbullying bystanders [11]. The majority of research examining students who witness cyberbullying, however, investigates why students do or do not intervene when they witness cyberbullying as bystanders [13,14,16,17,18,19,20,21,22], rather than examining the impact of observing cyberbullying on the bystanders themselves.

Although a growing body of literature documents the negative effects of witnessing traditional face-to-face bullying on bystanders [23,24,25,26], there is limited research examining mental health risks among students who witness cyberbullying [27]. When students who witness bullying do nothing to intervene, they may experience cognitive dissonance [28]. The dissonance between what students believe they should do (e.g., intervene to help the target of bullying) and what they actually do might account for mental health risks seen among bystanders [24,26]. Research examining the impact of school bullying on bystanders suggests that witnessing school bullying is associated with depression, anxiety [24,26], and somatic symptoms [26]. Researchers have also found that being exposed to school bullying as a bystander is related to feelings of isolation [23]. Further, when students witness bullying, they may feel anxious about becoming a target themselves or experience a degree of co-victimization [27]. Bystanders may also feel helpless [25], which can lead to passive behavior and contribute to depression, anxiety, and somatic symptoms. The feelings of helplessness, combined with cognitive dissonance related to remaining passive when witnessing bullying, may contribute to internalizing symptoms (i.e., depression, anxiety, and somatic symptoms) reported by students who witness school bullying.

It is unclear if the research demonstrating the relationship between internalizing symptoms and school bullying generalizes to cyberbullying. Although there are similarities between being a bystander of school bullying and cyberbullying, there are also some noteworthy differences. For example, in face-to-face bullying, bystanders are usually present, whereas in the case of cyberbullying, the bystander may witness the bullying while it is occurring or after the fact (e.g., a message is forwarded to them) [11]. Further, passive bystander behavior has been linked to moral disengagement [29,30], diffusion of responsibility [31], and a lack of confidence [32], knowledge, or skills to intervene [23,33]. These factors may play an even greater role in cyberbullying due to the lack of social–emotional cues [34,35], physical distance, real or perceived anonymity [34], and ease of disseminating communication via social networks [35]. In fact, research indicates witnessing cyberbullying is associated with higher levels of moral disengagement and lower feelings of responsibility and self-efficacy relative to witnessing traditional face-to-face bullying [35].

Despite the high prevalence rate of witnessing cyberbullying and the potential for negative outcomes for cyberbullying bystanders, we could find only one study examining the mental health risks associated with witnessing cyberbullying among middle school students [27]. Findings demonstrated witnessing cyberbullying was associated with higher rates of depression and anxiety. The authors, however, did not control for the effects of witnessing traditional school bullying. Research suggests that cyberbullying shares some common characteristics with school bullying and that there is considerable overlap between cyberbullying and school bullying [2,36,37]. Further, researchers have demonstrated a positive association between witnessing school bullying and depression and anxiety [24,26]. Thus, it is important to understand if internalizing symptoms is uniquely associated with witnessing cyberbullying or if the relationship is due to the overlap with witnessing school bullying. 

Although there is a large literature on the negative effects of being a target of cyberbullying [7,8,9] and a growing body of literature on the negative effects of witnessing school bullying [23,24,25,26], there is limited research on the relationship between witnessing cyberbullying and mental health risks among middle school students. Since school bullying and cyberbullying co-occur [2,36,37], it is important to examine the unique effects of witnessing cyberbullying on mental health risks. Additionally, because more than one half of middle school students witness cyberbullying [3], identifying the unique relationship between witnessing cyberbullying and mental health risks can help inform intervention programs for cyberbullying bystanders in this age group. 

### The Present Study

The purpose of this study is to address this gap by investigating the relationship between witnessing cyberbullying and internalizing symptoms among middle school students in the United States. Our aim was to examine if witnessing cyberbullying is associated with internalizing symptoms (i.e., depression, anxiety, and somatic symptoms) over and above the effects of witnessing traditional school bullying. Examining the relationship between witnessing cyberbullying and internalizing symptoms will extend the current research investigating mental health risks associated with cyberbullying victimization to cyberbullying bystanders. We hypothesized that cyberbullying bystanders would report higher levels of internalizing symptoms than non-bystanders over and above the effects of witnessing traditional school bullying. 

## 2. Materials and Methods

### 2.1. Participants

This study used a cross-sectional study design. We recruited middle school students from one public middle school in the Northwest region of the United States. Participants included 130 students in 6th–8th grade (57.4% female; 42.6% male). Ages ranged from 11 to 15 years old (*M* = 12.50 and *SD* = 1.00). The sample was predominantly White (58.5%) and Hispanic (36.9%).

### 2.2. Procedures

To match the demographic composition of the school, we used stratified proportionate sampling to randomly select 360 students. Three separate mailings were sent to parents/guardians (i.e., letter of introduction to the study, parental/guardian, consent, and a reminder letter). A project-addressed stamped envelope was provided to parents to send in the consent form. Letters were also provided to students to take home to their parents/guardians. All letters and consent forms were provided in Spanish and English. We received parental/guardian consent from 39.4% of students (N = 142). Of these, 130 provided student assent and 12 were absent from school on the day of data. The final response rate for this study was 36.1%. Parents of all participants gave their informed consent and all participants gave their informed assent for inclusion before they participated in the study. Students completed the research survey in the cafeteria at the school. Similar to other school-based intervention studies [38,39,40], participants were given pizza after data collection as an incentive. 

### 2.3. Measures

#### 2.3.1. Depression 

Depression was measured using the 12-item Depression Scale of the The Behavioral Assessment System for Children, Third Edition Self Report of Personality-Adolescent Form (BASC-3 SRP-A) [41]. Five items are rated on a dichotomous scale, 0 (*True*) or 2 (*False*). Example items include: “I don’t seem to do anything right”, “I just don’t care anymore”, and “I used to be happier”. Seven items are rated on a 4-point Likert scale ranging from 0 (*Never*) to 3 (*Almost Always*). Examples include: “I feel depressed”, “I feel life isn’t worth living”, and “I feel like I have no friends”. Reliability coefficients for the Depression Scale have been reported in the 0.80 range and have good construct validity, with correlations ranging from 0.51 to 0.93 with established measures of depression [41]. For this sample, Cronbach’s alpha was 0.92.

#### 2.3.2. Anxiety

Anxiety was measured using the 13-item Anxiety Scale of BASC-3 SRP-A [41]. Three items are rated on a dichotomous scale of 0 (*True*) or 2 (*False*). Example items include: “I can never seem to relax”, “I often worry about something bad happening to me”, and “I worry a lot of the time”. Ten items are rated on a 4-point Likert scale ranging from 0 (*Never*) to 3 (*Almost Always*). Examples include: “I feel anxious”, “I get so nervous I can’t breathe”, and “I worry when I go to bed at night”. The Anxiety Scale has reliability coefficient alphas ranging in the 0.80s for males and females and evidence of validity with correlations ranging from 0.50 to 0.97 between the Anxiety Scale and other established measures of anxiety [41]. Cronbach’s alpha for the sample in the current study was 0.88.

#### 2.3.3. Somatic Symptoms

Somatic symptoms were measured using the Somatic Complaints Subscale of the Child Behavioral Checklist (CBCL) [42]. The scale is comprised of 9 items assessing somatic complaints such as aches, pains, rashes, headaches, and stomachaches [42]. The items are rated on a 4-point Likert scale ranging from 0 (*Not True)* to 2 (*Very True or Often True*). Items are summed to create a total subscale score. The CBCL has established reliability and validity, with internal consistency of the Somatic Complaints Subscale reported at 0.78 [42]. For this sample, Cronbach’s alpha was 0.73.

#### 2.3.4. Bystander Status

Being a bystander of cyberbullying and traditional school bullying was measured using the question: “How often have you seen the following types of bullying in the past month?” The types of bullying listed were physical, verbal, relational, and cyberbullying. Examples of each type of bullying were provided. The items are rated on a 5-point Likert scale ranging from 0 (*Never*) to 4 (*Several Times a Day*). For cyberbullying bystander status, we dichotomized the cyberbullying scale to create bystander (>1) and non-bystander (0) status. Overall, 32.3% (*n* = 42) of students reported witnessing cyberbullying at least once in the past 30 days and were classified as bystanders. For witnessing school bullying, we combined the three school bullying items (i.e., physical, verbal, relational) to obtain the frequency of the witnessing school bullying variable (α = 0.75).

### 2.4. Data Analytic Plan

All analyses were conducted using SPSS version 25. We examined all variables to confirm that distributions had acceptable skew and kurtosis. We used a multivariate analysis of co-variance (MANCOVA) to examine effects of bystander status for the three outcome variables (depression, anxiety, somatic symptoms). We selected a MANCOVA because our research design is a between-groups design with one covariate and three outcome variables. ANCOVA is the appropriate analysis for between-groups designs with a covariate and the multivariate analysis controls for Type I errors. The fixed effect was bystander status (bystander; non-bystander). We conducted post hoc univariate analyses of variance (ANOVAs) to examine the significant effects. We included frequency of witnessing school bullying as a covariate in all analyses to assess if being a cyberbullying bystander is associated with internalizing symptoms over and above the effects of witnessing school bullying. Analyses were considered significant at *p* < 0.05. We used partial eta squared (η^2^*_p_*) as the measure of effect size with the magnitude as follows: small (η^2^*_p_* ≥ 0.01), medium (η^2^*_p_* ≥ 0.06), and large (η^2^*_p_* ≥ 0.14) [43,44]. 

### 2.5. Power Analysis

We conducted an a priori power analysis with the G*Power 3.1.3 program [45] to determine the adequate sample size to detect a medium effect size for each analysis. For the MANCOVA, the results of the power analysis indicated that a sample size of 180 is needed for a power of ≥0.80 to detect a medium effect size with an alpha level of 0.05. For the post hoc ANCOVAs, the results of the power analysis indicated that a sample size of 128 is needed for a power of ≥0.80 to detect a medium effect size with an alpha level of 0.05. As such, we randomly selected 360 with a projected response rate of 50% for a final sample of *N* = 180. Our actual response rate of 36.1% yielded a sample size of *N* = 130. Thus, we met the requirement for adequate power for the post hoc ANCOVAs to detect a medium effect size. However, with the sample size of 130, we were able to detect a large effect size with a power of >0.80 and a medium effect size with a power of >0.65, with an alpha level of 0.05, for the MANCOVA analysis.

### 2.6. Ethics Approval 

The protocol for this study was approved by the University Internal Review Board (107-SB16-242) and the school district, in accordance with the approved guidelines of the Declaration of Helsinki with written informed consent/assent from all participants (parents and their children).

## 3. Results

Means, standard deviations, and statistical contrast for the univariate ANCOVAs by bystander status are presented in Table 1. Results of the MANCOVA revealed a significant effect for cyberbullying bystander status, Wilks’ Lambda = 0.94, *F*(3, 125) = 2.82, *p* = 0.04, η^2^*_p_* = 0.07, when controlling for the effect of witnessing school bullying, Wilks’ Lambda = 0.89, *F*(3, 125) = 5.11, *p* = 0.01, η^2^*_p_* = 0.11. The effect size for bystander status is medium. Results indicate that the overall model is significant, with significant differences in outcomes between cyberbullying bystanders and non-bystanders over and above the effects of witnessing school bullying.

### 3.1. Depression

Post hoc ANCOVAs indicated a significant effect for cyberbullying bystander status for depression, *F*(1, 127) = 3.91, *p* < 0.05, η^2^*_p_* = 0.03, over and above the effect for witnessing school bullying, *F*(1, 127) = 0.95, *p* = 0.33, η^2^*_p_* = 0.01. The effect size was in the small to medium range. Examination of the means in Table 1 demonstrates that cyberbullying bystanders reported significantly higher levels of depression than cyberbullying non-bystanders. 

### 3.2. Anxiety

Post hoc ANCOVAs indicated a significant effect for cyberbullying bystander status for anxiety, *F*(1, 127) = 5.11, *p* < 0.02, η^2^*_p_* = 0.04, over and above the effect for witnessing school bullying, *F*(1, 127) = 6.10, *p* < 0.02, η^2^*_p_* = 0.05. The effect size was in the small to medium range. Examination of the means in Table 1 demonstrates that cyberbullying bystanders reported significantly higher levels of anxiety than cyberbullying non-bystanders. 

### 3.3. Somatic Complaints

Post hoc ANCOVAs indicated a significant effect for cyberbullying bystander status for somatic symptoms, *F*(1, 127) = 6.89, *p* < 0.01, η^2^*_p_* = 0.05, over and above the effect for witnessing school bullying, *F*(1, 127) = 13.90, *p* < 0.001, η^2^*_p_* = 0.10. The effect size was in the small to medium range. Examination of the means in Table 1 demonstrates that cyberbullying bystanders reported significantly higher levels of somatic symptoms than cyberbullying non-bystanders.

## 4. Discussion

Cyberbullying peaks in middle school, with more than 50% of middle school students reporting witnessing cyberbullying as bystanders in the past six months. Although the association between cyberbullying victimization and internalizing symptoms is well documented and research indicates witnessing traditional school bullying is associated with internalizing symptoms [24,26], there is limited research examining the impact of witnessing cyberbullying on bystanders [27]. Thus, the purpose of the present study was to address this gap by investigating the relationship between witnessing cyberbullying and internalizing symptoms among middle school students in the United States. We included witnessing school bullying as a covariate to control for the significant overlap between cyberbullying and school bullying [2,36]. Overall, our findings suggest that witnessing cyberbullying is uniquely associated with internalizing symptoms, including depression, anxiety, and somatic symptoms, even when controlling for the effect of witnessing traditional school bullying. 

Consistent with our hypothesis, results indicated that students who witness cyberbullying report significantly higher levels of depression, anxiety, and somatic symptoms than non-bystanders. Findings parallel prior research indicating witnessing cyberbullying is associated with depression and anxiety [27]. The current findings extend this literature by demonstrating that witnessing cyberbullying is associated with depression, anxiety, and somatic symptoms even after accounting for the effects of witnessing school bullying. This finding is consistent with research suggesting that being a target of cyberbullying is associated with internalizing symptoms over and above the effects of being a target of traditional bullying [10]. Thus, findings of this study add to the limited research suggesting that negative outcomes associated with cyberbullying extend beyond student targets to students who observe cyberbullying as bystanders.

There are several possible explanations for the association between witnessing cyberbullying and internalizing symptoms. For example, when students who witness bullying act passively, they may experience cognitive dissonance [28]. Bystanders may experience negative affective states related to the dissonance between believing they should intervene, but doing nothing to help the target [24]. Thus, cognitive dissonance may provide an explanation for internalizing symptoms reported by students who witness bullying [26]. Additionally, bystanders may feel helpless and anxious when they witness cyberbullying but do not know how to intervene. It is also possible that cyberbullying bystanders may have been targets themselves and may experience re-victimization or co-victimization [46] when observing others as targets of bullying. 

### 4.1. Limitations and Future Directions

Although this study adds to the sparse literature of the mental health risks for students who witness cyberbullying, some limitations deserve note. First, the sample was recruited from one middle school in the Northwest region of the United States and was relatively small. Although we recruited 360 students, we were only able to obtain parental consent from 39.4% parents and achieved a final response rate of 36.1%. Thus, our final sample was smaller than we expected leading to a reduction in power for our analyses. Although our MANCOVA analysis was underpowered for a medium effects size, we did achieve statistical significance for the model, allowing us to conduct the appropriately powered post hoc ANCOVA analyses. Additionally, the low response rate suggests that we may have nonresponse bias in our sample, which is often seen in research using active vs. passive parental consent procedures [47]. To improve the generalizability of the findings, larger samples from several schools should be used in future research. Next, because the study utilized a cross-sectional methodology, the causal direction of the relationship between witnessing cyberbullying and internalizing symptoms cannot be determined. Thus, longitudinal research is recommended for future studies. Further, we used a single item to measure witnessing cyberbullying. Using a multiple-item scale would improve the reliability and validity of the measure in future research. For example, a cyberbullying scale such as the Cyberbullying Questionnaire [48] could be modified with participants reporting if they witnessed the cyberbullying items (vs. experienced them as a target). Additionally, although we controlled for witnessing school bullying, we did not control for cyberbullying victimization as we did not collect those data as part of the current study. Since students who witness cyberbullying may also be targets if cyberbullying and victimization is related to mental health risks [7,8,9], future research should include cyberbullying victimization as a covariate. Finally, examining mediators of the relationship between bystander status and mental health risks was beyond the scope of this study. Future research could examine feelings of helplessness and cognitive dissonance as potential mediators of this relationship.

### 4.2. Implications for Practice 

Findings from this study reveal that 32.3% of students reported witnessing cyberbullying in the past 30 days. Thus, nearly one third of students may be experiencing depression, anxiety, and somatic symptoms related to witnessing cyberbullying. Mental health professionals inside and outside of the school setting need to understand that the impact of cyberbullying does not only affect targets of cyberbullying, but extends to those who witness cyberbullying as bystanders. Thus, it is imperative to address negative outcomes for middle students who witness cyberbullying as part of bullying prevention programs. 

Researchers have highlighted the importance of a systematic, whole-school approach to effectively prevent and manage all forms of bullying behavior, including cyberbullying [49]. However, according to a recent review of the cyberbullying intervention literature, the most frequently used intervention components included education on cyberbullying, coping skills and empathy training, communication and social skills, and digital citizenship [50]. These findings parallel traditional bullying programs, with research indicating that only a few school-wide, comprehensive bullying prevention programs include a bystander intervention component [51]. 

Researchers evaluating school-wide bullying prevention programs that include bystander intervention have demonstrated that these programs are effective in reducing cyberbullying [52], as well as reducing internalizing symptoms among students trained in the program [53]. Similarly, research indicates stand-alone bystander interventions are also effective in reducing internalizing symptoms for students trained to intervene in bullying situations [38,39,40,54]. Therefore, implementing comprehensive, school-wide interventions that include a bystander component [55] or brief, stand-alone bullying bystander interventions that focus specifically on bystander training for middle school students [56] may be a promising approach for reducing the mental health risks associated with witnessing cyberbullying.

## 5. Conclusions

This study is one of the first studies to examine the association between witnessing cyberbullying and internalizing symptoms among middle school students in the United States. Findings indicate that students who report witnessing cyberbullying as bystanders report higher levels of depression, anxiety, and somatic symptoms than non-bystanders. Moreover, these differences in internalizing symptoms were significant even when controlling for witnessing school bullying. Results underscore the importance of implementing bullying bystander interventions to reduce cyberbullying and the associated mental health risks for both targets of cyberbullying and students who witness cyberbullying as bystanders. 

## Figures and Tables

**Table 1:** Means, Standard Deviations, and Statistical Contrasts for Internalizing Symptoms by Bystander Status.

	Bystander		Non-Bystander				
Outcome	*M*	*SD*	*M*	SD	*F*(1,127)	*p*	η^2^*_p_*
Depression	7.91	8.42	4.92	6.45	3.91 *	0.05	0.03
Anxiety	13.82	8.50	9.56	7.61	5.61 *	0.02	0.04
Somatic Symptoms	4.48	3.43	2.76	2.40	5.92 **	0.01	0.05

* *p* < 0.05. ** *p* < 0.01.
